# Sex-Related Differences in Self-Reported Symptoms at Diagnosis in Pheochromocytomas and Paragangliomas

**DOI:** 10.1210/jendso/bvae005

**Published:** 2024-01-24

**Authors:** Stefanie Parisien-La Salle, Isabelle Bourdeau

**Affiliations:** Division of Endocrinology, Department of Medicine, Research Center, Centre Hospitalier de l’Université de Montréal (CHUM), Montreal, Quebec H2X 0A9, Canada; Division of Endocrinology, Department of Medicine, Research Center, Centre Hospitalier de l’Université de Montréal (CHUM), Montreal, Quebec H2X 0A9, Canada

**Keywords:** pheochromocytoma, paragangliomas, biological sex-related differences

## Abstract

**Context:**

Biological sex can play a role in the severity of certain diseases.

**Objective:**

Our objective was to evaluate whether sex-related differences affect the signs and symptoms of pheochromocytomas and paragangliomas (PPGLs) at presentation.

**Methods:**

We reviewed the records of patients with PPGLs at our center from 1995 to 2022.

**Results:**

Our study included 385 patients with PPGLs: 118 (30.6%) head and neck paragangliomas (HNPGLs), 58 (15.1%) thoracoabdominal paragangliomas (TAPGLs) and 209 (54.3%) pheochromocytomas (PHEOs). The cohort consisted of 234 (60.8%) women and 151 (39.2%) men. At diagnosis, more women than men presented with headaches (47.5% vs 32.4%; *P* = .007); however, more men presented with diabetes (21.1% vs 12.5%; *P* = .039). When subdivided by tumor location, headaches occurred more often in women with HNPGLs and TAPGLs (31.0% vs 11.4%; *P* = .0499 and 60.0% vs 21.7%; *P* = .0167). More men presented with diabetes among patients with PHEOs (28.2% vs 11.2%; *P* = .0038). In regard to nonsecretory PPGLs, women presented with a higher prevalence of headaches (46.9% vs 3.6%; *P* = .0002), diaphoresis (16.3% vs 0.0%; *P* = .0454), and palpitations (22.4% vs 0.0%; *P* = .0057). In patients with secretory tumors, women presented with more headaches (58.9% vs 42.7%; *P* = .0282) and men with more diabetes (29.3% vs 12.5%; *P* = .0035).

**Conclusion:**

In our cohort, more women presented with headaches across all tumor types and secretory statuses. More men presented with diabetes among patients with PHEOs and secretory tumors. In nonsecretory PPGLs, women had more adrenergic symptoms. These findings can be explained by differences in adrenergic receptor sensitivity, self-reported symptoms, and possibly other vasoactive peptides and sex-hormone status.

Males and females differ greatly in terms of physiology resulting in variations in disease presentation and incidence [1]. However, these distinctions have long been ignored in the field of research. Indeed, it was only in 1986 that the National Institutes of Health established a policy to encourage researchers to include women in studies [2]. In recent years, it has become very clear that biological sex must be included as an important factor influencing the prevalence, development, and outcome of disease [3, 4]. For example, males tend to have more aggressive disease in regard to papillary thyroid cancer (lymphovascular invasion and positive lymph nodes) [5], and female patients with diabetes tend to have a higher relative risk for vascular disease than males [6].

Pheochromocytomas (PHEOs) and paragangliomas (PGLs) (PPGLs) are rare neuroendocrine tumors that arise from the adrenal medulla (PHEOs) or from extra-adrenal chromaffin cells (PGLs) [7]. PPGLs can be functional when they oversecrete catecholamines, metanephrines, or methoxytyramine, or nonfunctional when there is an absence of catecholamines in tumor tissue [7, 8]. Catecholamines act on smooth muscles, blood vessels, and glands to increase blood pressure, increase heart rate, cause peripheral vasoconstriction, increase gluconeogenesis and glycolysis in the liver, and induce mydriasis and bronchodilatation [9].

The classic triad of hypertension, headaches, and diaphoresis associated with PPGLs carries a high specificity but is far from being present in all patients with these tumors [10, 11]. In a 2019 study by Geroula et al [10] comparing patients with and without PPGLs, the authors found that symptoms such as hyperhidrosis, palpitation, pallor, tremor, and nausea were more prevalent in patients with PPGLs, but headaches and flushing were not discriminatory. Many of these symptoms remain unspecific and carry a large array of differential diagnosis, ranging from menopause to Cushing syndrome to other neuroendocrine tumors [12, 13].

Studies exploring sex-related differences in PPGLs are scarce, with only 1 study in 2008 reporting on differences in the presentation of PHEOs between 23 males and 35 females [14]. More women than men were affected by self-reported symptoms (headaches [80% vs 52%], tremors [64% vs 33%], dizziness [83% vs 39%], anxiety [85% vs 50%], weight change [88% vs 43%], numbness [57% vs 24%], and changes in energy levels [89% vs 64%]) [14]. These findings suggest that sex-related differences may influence the nature of PPGLs that may affect optimal care/treatment and warrant a more thorough investigation. Thus, the objective of our study is to evaluate sex-related differences in the presentation and clinical sign and symptoms in a larger cohort of 385 patients with a variety of PPGLs.

## Materials and Methods

### Study Population

In this retrospective, single-center study, we reviewed the records of 385 patients with a diagnosis of PPGL at the Centre Hospitalier de l’Université de Montréal (CHUM) from January 1995 to January 2022. Information about biological sex, demographics, secretory status, and sign and symptoms at presentation were collected from each patient's chart. New standardized terminology for functionality status was used according to the 2022 systematic review by Constantinescu et al [8]. A patient with a nonsecretory tumor was defined as having normal plasma and/or urinary catecholamines. A secretory tumor was defined by plasma and/or urinary catecholamine levels above the upper limit of normal. A biochemically negative tumor was defined as normal plasma or urinary metanephrines and methoxytyramine [8]. It should be noted that plasmatic 3-metoxytyramine (3-MT) has been available at our center for only the last 5 years. Thus, we will use the terms *nonsecretory* (normal plasma and/or urinary catecholamines) and *secretory* (elevated plasma and/or urinary catecholamines) to define the hormonal status of PPGLs. Metastatic PPGLs were defined by presence of metastases in nonchromaffin tissues [7]. Patient symptoms were self-reported during patient interviews by physicians. Adrenergic symptoms were described as positive if they were chronic or paroxysmal in nature. Approval by the local ethics and research committees was obtained.

### Genetic Testing

From 2005 to 2015, genetic analysis included sequential gene testing and Multiplex Ligation-dependent Probe Amplication (MLPA) of specific genes (Molecular Diagnosis Laboratory of Alberta Health Services, Calgary, and Hôpital Européen Georges Pompidou Centre Georges Pompidou, Paris). Depending on when the genetic analysis was performed, the number of genes varied but mostly included the *RET*, *VHL*, and *SDHx* genes. From 2015 to 2020, a multigene panel was performed including at least 14 susceptibility genes: *SDHA*, *SDHAF2*, *SDHB*, *SDHC*, *SDHD*, *RET*, *VHL*, *FH*, *NF1*, *MAX*, *TMEM127*, *EGLN1*, *KIF1B*, and *MEN1* genes (Invitae, San Francisco) or *SDHA*, *SDHAF2*, *SDHB*, *SDHC*, *SDHD*, *RET*, *VHL*, *FH*, *NF1*, *MAX*, *TMEM127*, *EGLN1*, *ENGLN2*, *EPAS1*, and *MDH2* (Hôpital Européen Georges Pompidou Centre Georges Pompidou). Gene sequencing and deletion/duplication analysis were performed using next-generation sequencing technology for all genes except for the *SDHA* gene (Invitae), which was not evaluated for deletion/duplication. Genetic counseling was performed for each patient by a genetic counselor and written consent was obtained from each patient before genetic analysis.

### Statistical Analysis

Descriptive analyses included means and SDs for continuous variables and frequency of distribution for categorical variables. *T* tests are presented for variables consistent with a normal distribution. Chi-square or Fisher exact tests were conducted for categorical variables. Medians with interquartile range and Wilcoxon signed rank test are presented for continuous variables. Patients were stratified as male or female based on biological sex. Results were stratified by tumor location to account for differences in secretion by tumor type. All calculations were carried out with R version 4.2.1. As a primary analysis, we described signs and symptoms (hypertension, headaches, diaphoresis, palpitations, tremor, and presence of diabetes) at diagnosis according to biological sex and tumor location. We also described signs and symptoms at diagnosis depending on catecholamine profile that were subdivided into 3 groups: epinephrine/metanephrine (E/M), norepinephrine/normetanephrine (NE/NM), and metanephrine/normetanephrine (M/NM). We did not present the dopamine group as only 4 patients presented with an isolated dopamine secretion.

## Results

### Sex-Related Differences in the Pheochromocytomas and Paragangliomas Cohort

Our study included 385 patients with PPGLs: 118 (30.6%) head and neck paragangliomas (HNPGLs), 58 (15.1%) thoracoabdominal paragangliomas (TAPGLs), and 209 (54.3%) pheochromocytomas (PHEOs). The cohort consisted of 234 (60.8%) women and 151 (39.2%) men. Among the women (n = 234), 52.1% had PHEOs, 35.0% had HNPGLs, and 12.8% had TAPGLs. Of the men (n = 151), 57.6% had PHEOs, 23.8% had HNPGLs, and 18.5% had TAPGLs. Mean age was of 49.8 years (median: 50.5 years, minimum: 11 years, maximum: 88 years) and 80% were French Canadian. Overall, the most frequent presentation leading to diagnosis of PPGLs was incidental finding (incidentaloma) followed by mass effect. There was no statistically significant difference in regard to circumstances at diagnosis, tumor size, secretory status, metastatic status, or recurrences between sexes (Table 1).

**Table 1.** Clinicopathological characteristics of patients with pheochromocytomas and paragangliomas at diagnosis and follow-up separated by biological sex

	Female N = 234	Male N = 151	*P*
Mean age at diagnosis (SD; min-max), y	50.2 (15.6; 18-88)	49.1 (16.2; 11-84)	.482
PHEOs n, (%)	122 (52.1)	87 (57.6)	.**044**
HNPGLs n, (%)	82 (35.1)	36 (23.8)	
TAPGLs n, (%)	30 (12.8)	28 (18.5)	
Circumstances at diagnosis n, (%)			.478
Presymptomatic genetic testing	6 (2.7)	6 (4.0)	
Incidentaloma	85 (37.8)	66 (44.3)	
Mass effect	69 (30.7)	40 (26.8)	
Adrenergic symptoms	65 (28.9)	37 (24.8)	
Secretory at diagnosis n, (%)	125 (67.6)	103 (77.4)	.071
Tumor size at diagnosis median (IQR) (cm)	3.50 (2.50-5.50)	4.00 (2.50-6.35)	.140
Metastatic at diagnosis n, (%)	27 (12.4)	18 (12.3)	≥.999
Recurrence of PPGL during follow-up n, (%)	28 (14.9)	13 (9.8)	.24

Bold: *P* ≤ 0.05.

Circumstances at diagnosis: missing data for 9 women and 2 men; functionality: missing data for 49 women and 18 men; tumor size: missing data for 69 women and 32 men; metastatic at diagnosis: missing data for 16 women and 5 men; recurrence of PPGL during follow-up: missing data for 46 women and 19 men.

Abbreviations: HNPGLs, head and neck paragangliomas; IQR, interquartile range; max, maximum; min, minimum; PHEOs, pheochromocytomas; PPGLs, pheochromocytomas and paragangliomas; TAPGLs, thoracoabdominal paragangliomas.

At diagnosis, more women than men presented with headaches (47.5% vs 32.4%; *P* = .007). However, there was a higher prevalence of diabetes (21.1% vs 12.5%; *P* = .039) among men. Average body mass index for women was 25.45 and 26.64 in men (*P* = .952). Other adrenergic symptoms (hypertension, diaphoresis, tremor, and palpitations) did not have any statistically significant difference between the sexes (Table 2). To adjust for different secretory status patterns and tumor type, data were stratified by tumor location and secretory status.

**Table 2.** Signs and symptoms of pheochromocytomas and paragangliomas at presentation stratified by biological sex

Signs and symptoms	Female N = 234	Male N = 151	*P*
Hypertension at diagnosis n, (%)	133 (60.7)	91 (61.9)	.907
Headaches at diagnosis n, (%)	96 (47.5)	46 (32.4)	.**007**
Diaphoresis at diagnosis, n, (%)	67 (33.8)	46 (31.9)	.802
Palpitation at diagnosis n, (%)	89 (43.8)	52 (36.1)	.182
Tremor at diagnosis n, (%)	25 (14.6)	17 (13.9)	≥.999
Diabetes at diagnosis n, (%)	28 (12.5)	31 (21.1)	.**039**

Bold: *P* ≤ 0.05.

Hypertension at diagnosis: missing data for 15 women and 4 men; headaches at diagnosis: missing data for 32 women and 9 men; diaphoresis at diagnosis: missing data for 36 women and 7 men; palpitation at diagnosis: missing data for 31 women and 7 men; tremor at diagnosis: missing data for 63 women and 29 men; diabetes at diagnosis: missing data for 10 women and 4 men.

### Sex-Related Differences in Pheochromocytomas

Within our cohort dating from 1995 to 2022, more women than men were diagnosed with PHEOs (58.4% vs 41.6%). Men with PHEOs were more likely to present with diabetes at the time of tumor diagnosis (28.2% vs 11.2%; *P* = .0038). There was no statistically significant difference between the sexes for hypertension, headaches, diaphoresis, palpitations, or tremors (Table 3).

**Table 3.** Clinical signs and symptoms of pheochromocytomas and paragangliomas stratified by biological sex and tumor type

Signs and symptoms	PHEOs N = 209			HNPGLs N = 118			TAPGLs N = 58		
	Female n = 122 (58.4%)	Male n = 87 (41.6%)	*P*	Female n = 82 (69.5%)	Male n = 36 (30.5%)	*P*	Female n = 30 (51.7%)	Male n = 28 (48.3%)	*P*
Hypertension n, (%)	79 (69.9)	64 (74.4)	.5883	31 (39.2)	8 (22.2)	.1152	23 (85.2)	19 (76.0)	.4922
Headaches n, (%)	59 (55.7)	37 (44.0)	.1488	22 (31.0)	4 (11.4)	.**0499**	15 (60.0)	5 (21.7)	.**0167**
Diaphoresis n, (%)	50 (49.0)	36 (42.4)	.4452	9 (12.7)	1 (2.9)	.1599	8 (32.0)	9 (37.5)	.9171
Palpitation n, (%)	62 (57.9)	45 (52.9)	.5844	12 (16.9)	0 (0.0)	.**0079**	15 (60.0)	7 (29.2)	.0598
Tremor n, (%)	20 (21.7)	15 (20.3)	.9687	1 (1.8)	0 (0)	≥.999	4 (17.4)	2 (9.1)	.6652
Diabetes n, (%)	13 (11.2)	24 (28.2)	.**0038**	9 (11.5)	2 (5.6)	.498	6 (20.0)	5 (19.2)	≥.999

Bold: *P* ≤ 0.05.

PHEOs: Hypertension at diagnosis: missing data for 9 women and 1 man; headaches at diagnosis: missing data for 16 women and 3 men; diaphoresis at diagnosis: missing data for 20 women and 2 men; palpitation at diagnosis: missing data for 15 women and 2 men; tremor at diagnosis: missing data for 30 women and 13 men; diabetes at diagnosis: missing data for 6 women and 2 men.

HNPGLs: Hypertension at diagnosis: missing data for 3 women and 0 men; headaches at diagnosis: missing data for 11 women and 1 man; diaphoresis at diagnosis: missing data for 11 women and 1 man; palpitation at diagnosis: missing data for 11 women and 1 man; tremor at diagnosis: missing data for 26 women and 10 men; diabetes at diagnosis: missing data for 4 women and for 0 men.

TAPGLs: Hypertension at diagnosis: missing data for 3 women and 3 men; headaches at diagnosis: missing data for 5 women and 5 men; diaphoresis at diagnosis: missing data for 5 women and 4 men; palpitation at diagnosis: missing data for 5 women and 4 men; tremor at diagnosis: missing data for 7 women and 6 men; diabetes at diagnosis: missing data for 0 women and 2 men.

Abbreviations: HNPGLs, head and neck paragangliomas; PHEOs, pheochromocytomas; TAPGLs, thoracoabdominal paragangliomas.

### Sex-Related Differences in Head and Neck Paragangliomas

More women than men were diagnosed with HNPGLs (69.5% vs 30.5%). More women also presented with hypertension (39.2% vs 22.2%), headaches (31.0% vs 11.4%), diaphoresis (12.7% vs 2.9%), and palpitations (16.9% vs 0.0%). The association between sexes was statistically significant for headaches (*P* = .0499) and palpitations (*P* = .0079) (see Table 3).

### Sex-Related Differences in Thoracoabdominal Paragangliomas

The incidence of TAPGLs was slightly higher in women than in men (51.7% vs 48.3%). Women were more likely than their male counterparts to present with headaches (60.0 vs 21.7%) and palpitations (60.0 vs 29.2%) (see Table 3). This association was statistically significant for headaches (*P* = .0167) (see Table 3).

### Sex-Related Differences According to Secretory Status and Type of Catecholamines

#### Secretory tumors

More women with secretory tumors presented with headaches (58.9% vs 42.7%; *P* = .0282) than their male counterparts. When subdivided by catecholamine profile, the higher frequency in women was present in the NE/NM group (56.9% vs 37%) and in the M/NM group (70.7% vs 55.9%). More men with secretory tumors had diabetes at diagnosis than women (29.3% vs 12.5%; *P = .035*). This higher frequency was present in every type of catecholamine profile and secretory status (Tables 4 and 5).

**Table 4.** Clinical signs and symptoms of pheochromocytomas and paragangliomas stratified by biological sex and secretory status

Signs and symptoms	Nonsecretory N = 90			Secretory N = 228		
	Female n = 60 (66.7%)	Male n = 30 (33.3%)	*P*	Female n = 125 (54.8%)	Male n = 103 (45.2%)	*P*
Hypertension n, (%)	26 (45.6)	9 (30.0)	.2373	85 (70.8)	75 (75.8)	.5064
Headaches n, (%)	23 (46.9)	1 (3.6)	.**0002**	66 (58.9)	41 (42.7)	.**0282**
Diaphoresis n, (%)	8 (16.3)	0 (0.0)	.**0454**	55 (49.5)	46 (46.9)	.8117
Palpitation n, (%)	11 (22.4)	0 (0.0)	.**0057**	69 (61.1)	51 (52.0)	.2379
Tremor n, (%)	1 (2.1)	0 (0.0)	≥.999	23 (23.5)	17 (20.0)	.6987
Diabetes n, (%)	8 (13.8)	2 (6.7)	.4838	15 (12.5)	29 (29.3)	.**0035**

Bold: *P* ≤ 0.05.

Secretory status missing for 67 patients.

Nonsecretory: Hypertension at diagnosis: missing data for 3 women and 0 men; headaches at diagnosis: missing data for 11 women and 2 men; diaphoresis at diagnosis: missing data for 11 women and 2 men; palpitation at diagnosis: missing data for 11 women and 2 men; tremor at diagnosis: missing data for 13 women and 7 men; diabetes at diagnosis: missing data for 2 women and 0 men.

Secretory: Hypertension at diagnosis: missing data for 5 women and 4 men; headaches at diagnosis: missing data for 13 women and 7 men; diaphoresis at diagnosis: missing data for 14 women and 5 men; palpitation at diagnosis: missing data for 12 women and 5 men; tremor at diagnosis: missing data for 27 women and 18 men diabetes at diagnosis: missing data for 0 women and 4 men.

**Table 5.** Clinical signs and symptoms of pheochromocytomas and paragangliomas stratified by biological sex and catecholamine profile

Signs and symptoms	E/M N = 23		NE/NM N = 109		M/NM N = 82	
	Female n = 12 (52.2%)	Male n = 11 (47.8%)	Female n = 58 (53.2%)	Male n = 51 (46.8%)	Female n = 47 (57.3%)	Male n = 35 (42.7%)
Hypertension n, (%)	4 (33.3)	7 (70.0)	40 (74.1)	39 (79.6)	35 (76.1)	26 (76.5)
Headaches n, (%)	3 (25.0)	3 (30.0)	29 (56.9)	17 (37.0)	29 (70.7)	19 (55.9)
Diaphoresis n, (%)	6 (50.0)	5 (50.0)	23 (45.1)	23 (48.9)	21 (52.5)	16 (45.7)
Palpitation n, (%)	6 (54.5)	5 (50.0)	30 (56.6)	24 (51.1)	28 (68.3)	21 (60.0)
Tremor n, (%)	3 (27.3)	4 (40.0)	10 (21.7)	2 (5.0)	8 (24.2)	11 (37.9)
Diabetes n, (%)	0 (0.0)	3 (27.3)	10 (18.2)	14 (29.8)	5 (10.9)	11 (31.4)

Catecholamine profile missing for 14 patients.

E/Ms: Hypertension at diagnosis: missing data for 0 women and 1 man; headaches at diagnosis: missing data for 0 women and 1 man; diaphoresis at diagnosis: missing data for 0 women and 1 man; palpitation at diagnosis: missing data for 1 woman and 1 man; tremor at diagnosis: missing data for 1 woman and 1 man; diabetes at diagnosis: missing data for 1 woman and 0 men.

NE/NM: Hypertension at diagnosis: missing data for 4 women and 2 men; headaches at diagnosis: missing data for 7 women and 5 men; diaphoresis at diagnosis: missing data for 7 women and 4 men; palpitation at diagnosis: missing data for 5 women and 4 men; tremor at diagnosis: missing data for 12 women and 11 men; diabetes at diagnosis: missing data for 3 women and 4 men.

M/NM: Hypertension at diagnosis: missing data for 1 woman and 1 man; headaches at diagnosis: missing data for 6 women and for 1 man; diaphoresis at diagnosis: missing data for 7 women and 0 men; palpitation at diagnosis: missing data for 6 women and 0 men; tremor at diagnosis: missing data for 14 women and 6 men; diabetes at diagnosis: missing data for 1 woman and 0 men.

Abbreviations: E/M, epinephrine/metanephrine; M/NM, metanephrine/normetanephrine; NE/NM, norepinephrine/normetanephrine.

#### Nonsecretory tumors

In regard to nonsecretory PPGLs, more women presented with adrenergic symptoms: headaches (46.9% vs 3.6%), diaphoresis (16.3% vs 0.0%), and palpitations (22.4% vs 0.0%). These were all statistically significant. They were also associated with a higher prevalence of hypertension (45.6% vs 30.0%) and diabetes (13.8% vs 6.7%) but these associations were not statistically significant (see Table 4).

### Sex-Related Differences According to Age

To take into account the effect of age on the development of certain signs and symptoms, we divided the group of men and women into 2 groups: younger than 50 years and 50 years and older. These groups were very similar in terms of number of patients: younger than 50 years (N = women: 112, men: 70) and 50 years and older (N = women: 117, men: 75).

#### Men and women younger than 50 years

In this subgroup we found the same associations as in our whole cohort. Women presented with more headaches than men (57.7% vs 45.2%), and men presented with more diabetes than women (10.6% vs 3.8%). However, in both cases, these associations were not statistically significant.

#### Men and women aged 50 years and older

The prevalence of hypertension and diabetes was higher in the group of men and women aged 50 years and older (diabetes: women: 21.2%, men: 30.7% ; hypertension: women: 69.7%, men: 72.2%) compared to the younger than 50 years group (diabetes: women: 3.8%, men: 10.6% ; hypertension: women: 50.1%, men: 47.8%), as expected [15].

Interestingly, older men and women presented with a lower prevalence of headaches than the younger group. Indeed, women aged 50 years and older presented with headaches in 35.6% of cases compared to men in 20.8%. This association was statistically significant.

### Genotype Distribution According to Sex

In regard to genotype, the only difference we observed was a higher prevalence of *SDHD* mutations in women compared to men (3.7% vs 0%). Interestingly, all 5 germline *SDHD* mutations were identified in women with nonfunctional HNPGLs. Eighty percent had headaches, 20% had diaphoresis, and 20% had palpitations. Also, *SDHC* and *MAX* pathogenic variants were more prevalent in men (11.4% vs 7.5% and 3.4% vs 0.7%, respectively) (Table 6).

**Table 6.** Genotype distribution according to sex

	Genetic results (n = 222)			
	Men (n = 88)		Women (n = 134)	
	No.	%	No.	%
No mutation	49	55.7	81	60.4
*VUS*	5	5.7	10	7.5
*SDHA*	1	1.1	2	1.5
*SDHB*	6	6.8	5	3.7
*SDHC*	10	11.4	10	7.5
*SDHD*	0	0.0	5	3.7
*NF1*	4	4.5	8	6.0
*MAX*	3	3.4	1	0.7
*RET*	6	6.8	7	5.2
*VHL*	4	4.5	3	2.2
*FH*	0	0.0	2	1.5

Abbreviation: VUS, variant of unknown significance.

## Discussion

We present the largest study evaluating sex-related differences in signs and symptoms of PPGLs at diagnosis in a cohort of Canadian patients. Our cohort was represented by more women than men (60.8% vs 39.2%), which may suggest a higher prevalence of PPGL among women. Our numbers are similar to a large Italian cohort of 501 patients with PPGLs, among whom 57.5% were female [16]. In our cohort, when subdivided by tumor location, the female predominance was mainly driven by HNPGLs and PHEOs; almost 70% of patients with HNPGLs were women and almost 60% of patients with PHEOs were women. Other studies have also shown that HNPGLs may be predominant in women. For example, in a cohort of 54 HNGPLs described by Singh et al, 64.8% of patients were female [17].

The pathogenesis of headaches related to PHEOs remains unclear. The Headache Classification Committee of the International Headache Society classifies headaches associated with PHEOs under “headache attributed to arterial hypertension” [18]. However, cases of severe headaches associated with PPGLs in normotensive patients have been described [19, 20], suggesting that hypertension may not be the only contributor to headaches and PPGLs. The type of headache can also vary in its prevalence within the general population [21]. Tension-type headaches are 1.5 times more frequent in women, whereas cluster headaches are more common in young men, and migraines seem to be 2 to 3 times more common in postpubertal women than in men [21]. Variations in estrogen are also known to contribute to the pathogenesis of migraines [22]. In our study, more women than men presented with headaches (47.5% vs 32.4%; *P* = .007) across all tumor types but was statistically significant only in HNPGLs (31.0% vs 11.4%) and TAPGLs (60.0% vs 21.7%). In our study, more women than men presented with headaches (47.5% vs 32.4%; *P* = .007) across all tumor types but was statistically significant only in HNPGLs (31.0% vs 11.4%) and TAPGLs (60.0% vs 21.7%). This is a higher prevalence than what is described for migraines (20.7% of women and 9.7% of men) [23] and tension headaches (25% vs 19%) [24] in the general population, suggesting that PPGL-related headaches are a different entity that are also associated with a higher prevalence in women than in men. In regard to secretory status, more women presented with headaches in the presence of secretory tumors (58.9% vs 42.7%; *P* = .0282) and nonsecretory tumors (46.9% vs 3.6%; *P* = .0002). This led us to believe that the higher prevalence of headaches in women was not because of a difference in catecholamines sensitivity or subtype. A study by Lance and Hinterberger [25] on the relationship between subtype of catecholamines and PHEO symptoms concluded that headaches did not have any relationship to the ratio of NE to E. Another hypothesis on the pathogenesis of headaches in PPGLs is the secretion of other vasoactive peptides by PPGLs, including adrenomedullin and calcitonin gene-related peptide, both of which could have a causative role in episodic vascular headaches [20, 26]. A murine study demonstrated that female sex steroids increase adrenomedullin-induced vasodilation by increasing the expression of adrenomedullin receptor components in the rat mesenteric artery [27]. When subdividing by age group, women always presented more headaches than men; however, this association was statistically significant only in the aged 50 and older group. Interestingly, the prevalence of headaches was higher in men and women younger than 50 years compared to their older counterparts. Yet, the sex difference seems most apparent after age 50 years. Recently, a team led by Ross et al [28] has shed some light on the role of G protein–coupled receptors in the pathogenesis of PPGLs. The authors demonstrated in vitro that PHEOs can express luteinizing hormone (LH) chorionic gonadotropin receptors and can have human chorionic gonadotropin–mediated E secretion [28]. It is hypothesized that the higher LH levels in postmenopausal women could trigger adrenergic crisis [28]. Mean age at diagnosis for women with PPGLs in our cohort was 50.2 years, which is close to the mean age of menopause. We can speculate that these receptors could also be part of the reason why women present with more headaches than men. Indeed, the LH chorionic gonadotropin receptor may be responsible for mediating other hormone secretions that could explain the sex-related differences found in our study.

In HNPGLs, more women than men presented with palpitations (16.9% vs 0%; *P* = .0079). Of the 118 HNPGLs in our cohort, secretory status at diagnosis was known for 75 patients. Of these, 68 were nonsecretory (90.7%). It should be noted that 3-methoxytyramine (3-MT) measurements have been accessible at our center only for the past 5 years; thus, most HNPGLs in our cohort were not tested for 3-MT secretion at initial diagnosis. Van Duinen et al [29] described a cohort of 136 patients with HNPGLs, of which 23% had urinary 3-MT excess and were associated with more palpitations and diaphoresis, collapse, and a higher pulse rate compared to nonsecretory HNPGLs. In addition, 61% of these patients were female and 39% were male, whereas 57% of the patients without 3-MT excess were male and 43% were female. The sex-related difference associated with 3-MT excess was shown to be statistically significant. Thus, this study shows that women with HNPGLs presented with more 3-MT excess and associated symptoms than their male counterparts [29]. As we did not screen our patients for 3-MT excess, we can only hypothesize that this could explain why female patients with HNPGLs presented with more palpitations than their male counterparts in our cohort. However, palpitations are not specific of PPGLs and confounding factors such as anxiety cannot be definitively excluded as contributors to this observation.

According to Statistics Canada, the prevalence of diabetes in Canadian adults was 10% in men and 7% in women from 2016 to 2019 [30]. The prevalence of glucose intolerance and diabetes in PHEOs is between 35% and 55% [31]. This is due to increased peripheral insulin resistance, excess glucagon secretion, and insulin inhibition due to catecholamine excess [31]. β-Adrenergic receptors are responsible for glycogenolysis and glucagon secretion and α-adrenergic receptors for inhibiting insulin secretion [32, 33]. In general, diabetes is more prevalent in male [34] than female patients, due in part to the protective role of estrogen on energy expenditure, insulin sensitivity, and adipose tissue distribution [35]. In our study, more men presented with diabetes at diagnosis than women (21.1% vs 12.5%), with a higher frequency than expected in the general Canadian population, supporting a link between PPGL and the development of diabetes in men, which was not the case in women. When subdivided by tumor location, this association was driven by PHEOs, where 28.2% of men had diabetes at diagnosis compared to 11.2% of women. This association was also found in secretory tumors (29.3% vs 12.5%) and was present in every type of catecholamine profile, but was most evident in the E/M group. This is not surprising since epinephrine-secreting PHEOs are more likely to produce glucose disturbances due to their higher affinity for the β2-adrenergic receptors [33]. This association was not found in nonsecretory tumors, suggesting that the PHEO association with diabetes is catecholamine driven.

The main limitation of our study was its retrospective nature. This limited the data available, such as initial biochemical status that included plasmatic 3-MT, which has been available at our center only for the last 5 years. Most symptoms are qualitative in nature, with patients reporting symptoms in response to a physician questionnaire. This can lead to response bias. According to the literature, female patients tend to have more self-reported symptoms [36]. Moreover, our secondary analysis (sex-related differences adjusted for secretory status) exposes us to multiple comparison bias and to spurious findings. Finally, this study has some missing data, which may have subtle effects on the precision and generalizability of our findings.

In summary, this study provides insight about the sex-related differences in the signs and symptoms of PPGLs. In our cohort, women were more likely than men to present with headaches in all tumor types, including secretory and nonsecretory tumors. Men were more likely to present with diabetes in association with PHEOs and secretory tumors. Among those with nonsecretory tumors, women had more adrenergic symptoms at presentation (headache, diaphoresis, and palpitations). These findings are most likely multifactorial and can be explained by differences in adrenergic receptor sensitivity, self-reported symptoms, and possibly other vasoactive peptides and sex-hormone status. More studies are needed to elucidate these associations.

## Data Availability

Some or all data sets generated during and/or analyzed during the current study are not publicly available but are available from the corresponding author on reasonable request.

## Abbreviations

3-MT: 3-metoxytyramine
E/M: epinephrine/metanephrine
HNPGLs: head and neck paragangliomas
LH: luteinizing hormone
M/NM: metanephrine/normetanephrine
NE/NM: norepinephrine/normetanephrine
PGLs: paragangliomas
PHEOs: pheochromocytomas
PPGLs: pheochromocytomas and paragangliomas
TAPGLs: thoracoabdominal paragangliomas
